# *catena*-Poly[disilver(I)(*Ag*–*Ag*)-bis­(μ_3_-quinoline-3-carboxyl­ato)-1:2:1′κ^3^ *O*:*O*′:*N*;2:1′′:2′′κ^3^ *N*:*O*:*O*′]

**DOI:** 10.1107/S1600536812035209

**Published:** 2012-08-15

**Authors:** Chun-Bo Liu, Yao Cong, He-Yi Sun

**Affiliations:** a School of Chemistry and Chemical Engineering, Jiangsu University, Zhenjiang 212013, People’s Republic of China

## Abstract

In the title compound, [Ag_2_(C_10_H_6_NO_2_)_2_]_*n*_, the Ag^I^ atom is coordinated by one N atom and two O atoms from three quinoline-3-carboxyl­ate ligands in a T-shaped fashion, with an additional Ag⋯Ag distance of 2.9468 (6) Å. The ligands connect the Ag^I^ atoms into a double-chain structure along [010]. Weak Ag⋯O inter­actions [Ag⋯O = 2.802 (3) and 2.877 (4) Å] link the double-chains into a layer network parallel to (101). π–π inter­actions are also observed in the layer network [centroid–centroid distances = 3.780 (3) and 3.777 (3) Å].

## Related literature

For background to the design and applications of structures with metal-organic frameworks and of Ag^I^ complexes, see: Sun *et al.* (2010 ▶); Wei *et al.* (2006 ▶); Yilmaz *et al.* (2008 ▶). For related structures, see: Baenziger *et al.* (1986 ▶); Yang *et al.* (2004 ▶); Yeşiilel *et al.* (2011 ▶); You *et al.* (2004 ▶).
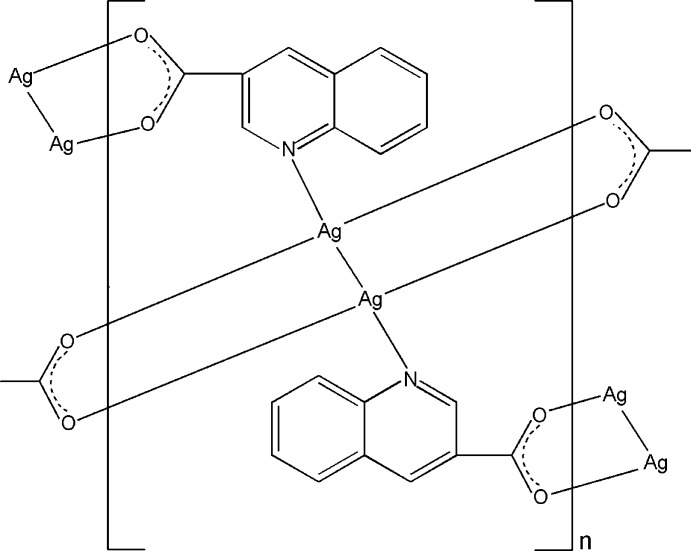


## Experimental

### 

#### Crystal data

[Ag_2_(C_10_H_6_NO_2_)_2_]
*M* *_r_* = 560.06
Triclinic, 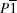

*a* = 8.0583 (15) Å
*b* = 8.4824 (15) Å
*c* = 12.934 (2) Å
α = 93.225 (2)°
β = 94.812 (2)°
γ = 104.640 (2)°
*V* = 849.6 (3) Å^3^
*Z* = 2
Mo *K*α radiation
μ = 2.34 mm^−1^
*T* = 293 K
0.13 × 0.11 × 0.10 mm

#### Data collection

Bruker APEX CCD diffractometer
Absorption correction: multi-scan (*SADABS*; Sheldrick, 1996 ▶) *T*_min_ = 0.745, *T*_max_ = 0.792
6197 measured reflections
2962 independent reflections
2298 reflections with *I* > 2σ(*I*)
*R*_int_ = 0.027

#### Refinement

*R*[*F* ^2^ > 2σ(*F* ^2^)] = 0.030
*wR*(*F* ^2^) = 0.081
*S* = 1.01
2962 reflections
253 parameters
168 restraints
H-atom parameters constrained
Δρ_max_ = 0.51 e Å^−3^
Δρ_min_ = −0.52 e Å^−3^

### 

Data collection: *SMART* (Bruker, 2007 ▶); cell refinement: *SAINT* (Bruker, 2007 ▶); data reduction: *SAINT*; program(s) used to solve structure: *SHELXS97* (Sheldrick, 2008 ▶); program(s) used to refine structure: *SHELXL97* (Sheldrick, 2008 ▶); molecular graphics: *XP* in *SHELXTL* (Sheldrick, 2008 ▶) and *DIAMOND* (Brandenburg, 1999 ▶); software used to prepare material for publication: *SHELXTL*.

## Supplementary Material

Crystal structure: contains datablock(s) global, I. DOI: 10.1107/S1600536812035209/hy2558sup1.cif

Structure factors: contains datablock(s) I. DOI: 10.1107/S1600536812035209/hy2558Isup2.hkl

Additional supplementary materials:  crystallographic information; 3D view; checkCIF report

## Figures and Tables

**Table 1:** Selected bond lengths (Å)

Ag1—N1	2.429 (3)
Ag1—O2^i^	2.219 (3)
Ag1—O4^ii^	2.220 (3)
Ag2—N2	2.373 (3)
Ag2—O1^i^	2.282 (3)
Ag2—O3^ii^	2.258 (3)

Symmetry codes: (i) 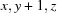; (ii) 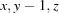.

**Fig. 1.**
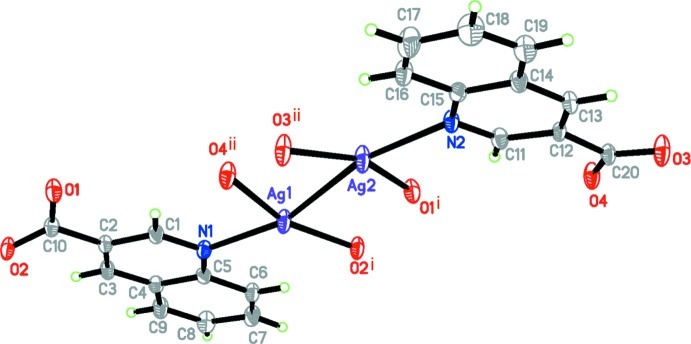
The asymmetric unit of the title compound. Displacement ellipsoids are drawn at the 30% probability level. [Symmetry codes: (i) x, y+1, z; (ii) x, y-1, z.]

**Fig. 2.**
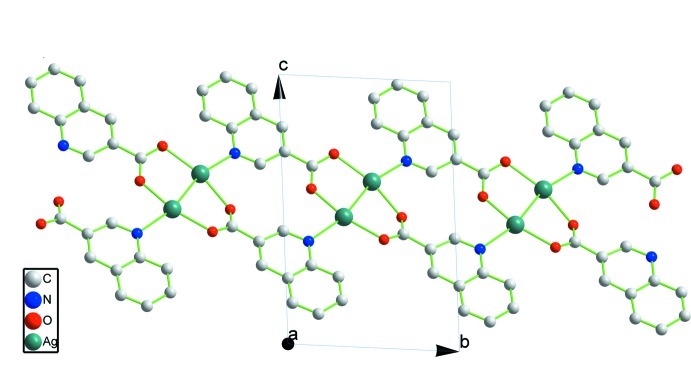
The one-dimensional double-chain of the title compound. H atoms have been omitted for clarity.

## supplementary crystallographic information

### Comment

In recent years, the design and synthesis of metal-organic frameworks (MOFs) based on assembly of suitable and rigid building blocks have attracted great attention for their interesting structures and potential applications in catalysis, separation, gas storage and molecular recognition (Wei *et al.*, 2006). Moreover, Ag(I) ion is easy to form short Ag–Ag contacts as well as ligand unsupported interactions, which have been proved to be two of the most important factors contributing to the formation of such complexes and special properties (Yilmaz *et al.*, 2008). Much attention has been paid to Ag(I) ion as its d^10^ closed-shell electronic configuration. It demonstrates a dynamic range of coordinative geometries, including linear, trigonal-planar, tetrahedral and trigonal-pyramidal. In occasional, it also has examples of square-planar, pyramidal and octahedral geometries, and a tendency to form an argentophilic interaction, both of which may lead to discovery of novel structural motifs (Sun *et al.*, 2010). It is well known that quinoline-3-carboxylic acid (H*L*) acts as a polyfunctional ligand in metal complexes and coordinates to metals by means of its carboxylate oxygen and a nitrogen atom, exhibiting different coordination modes, such as monodentate-N and monodentate-O, bis(monodentate), bidentate(N, O) and bridging form. In addition, H*L* also displays an extend π-system, which is beneficial for the formation of π–π interactions to generate high dimensional supramolecular architectures and further stabilize the network. Therefore, we selected silver ion and H*L* to obtain the title compound under hydrothermal conditions.

In the title compound, the Ag^I^ is coordinated by one N atom and two O atoms from three *L* ligands (Fig. 1, Table 1) and also forms an Ag···Ag contact (Baenziger *et al.*, 1986; Yang *et al.*, 2004). The distance of Ag1···Ag2 is 2.9468 (6) Å. It is shorter than the sum of the van der Waals radii of two silver(I) atoms (3.44 Å), thus the Ag—Ag interaction is found (Yeşilel *et al.*, 2011; You *et al.*, 2004). The bidentate bridging carboxylate group of the ligand connect two Ag atoms and the pyridine N atom links another Ag atom, leading to the formation of a one-dimensional double-chain structure (Fig. 2). The weak Ag···O interactions, with Ag1···O2^i^ and Ag2···O1^ii^ distances of 2.802 (3) and 2.877 (4) Å [symmetry codes: (i) 2-x, -y, 1-z; (ii) 1-x, -y, 1-z], link the double-chains into a layer network. π–π interactions are observed in the layer network [centroid–centroid distances = 3.780 (3) and 3.777 (3) Å].

### Experimental

H*L* was purchased commercially and used without further purification. A mixture of AgCl (14.33 mg, 0.1 mmol) and H*L* (17.30 mg, 0.1 mmol) was dissolved in a 10 ml of water with pH = 6. The resulting mixture was heated in a 15 ml Teflon-lined autoclave at 438 K for three days. Then the autoclave was slowly cooled to room temperature and colourless block-shaped crystals were obtained in a yield of 50%.

### Refinement

H atoms were positioned geometrically and refined as riding atoms, with C—H = 0.93 Å and with *U*_iso_(H) = 1.2*U*_eq_(C).

### Crystal data

**Table:**

[Ag_2_(C_10_H_6_NO_2_)_2_]	*Z* = 2
*M**_r_* = 560.06	*F*(000) = 544
Triclinic, *P*1	*D*_x_ = 2.189 Mg m^−^^3^
Hall symbol: -P 1	Mo *K*α radiation, λ = 0.71073 Å
*a* = 8.0583 (15) Å	Cell parameters from 7499 reflections
*b* = 8.4824 (15) Å	θ = 1.6–27.5°
*c* = 12.934 (2) Å	µ = 2.34 mm^−^^1^
α = 93.225 (2)°	*T* = 293 K
β = 94.812 (2)°	Block, colourless
γ = 104.640 (2)°	0.13 × 0.11 × 0.10 mm
*V* = 849.6 (3) Å^3^	

### Data collection

**Table:**

Bruker APEX CCD diffractometer	2962 independent reflections
Radiation source: fine-focus sealed tube	2298 reflections with *I* > 2σ(*I*)
Graphite monochromator	*R*_int_ = 0.027
φ and ω scans	θ_max_ = 25.0°, θ_min_ = 1.6°
Absorption correction: multi-scan (*SADABS*; Sheldrick, 1996)	*h* = −9→9
*T*_min_ = 0.745, *T*_max_ = 0.792	*k* = −10→10
6197 measured reflections	*l* = −15→13

### Refinement

**Table:**

Refinement on *F*^2^	Primary atom site location: structure-invariant direct methods
Least-squares matrix: full	Secondary atom site location: difference Fourier map
*R*[*F*^2^ > 2σ(*F*^2^)] = 0.030	Hydrogen site location: inferred from neighbouring sites
*wR*(*F*^2^) = 0.081	H-atom parameters constrained
*S* = 1.01	*w* = 1/[σ^2^(*F*_o_^2^) + (0.0428*P*)^2^] where *P* = (*F*_o_^2^ + 2*F*_c_^2^)/3
2962 reflections	(Δ/σ)_max_ = 0.001
253 parameters	Δρ_max_ = 0.51 e Å^−^^3^
168 restraints	Δρ_min_ = −0.52 e Å^−^^3^

### Special details

**Table:**

Geometry. All esds (except the esd in the dihedral angle between two l.s. planes) are estimated using the full covariance matrix. The cell esds are taken into account individually in the estimation of esds in distances, angles and torsion angles; correlations between esds in cell parameters are only used when they are defined by crystal symmetry. An approximate (isotropic) treatment of cell esds is used for estimating esds involving l.s. planes.
Refinement. Refinement of F^2^ against ALL reflections. The weighted R-factor wR and goodness of fit S are based on F^2^, conventional R-factors R are based on F, with F set to zero for negative F^2^. The threshold expression of F^2^ > 2sigma(F^2^) is used only for calculating R-factors(gt) etc. and is not relevant to the choice of reflections for refinement. R-factors based on F^2^ are statistically about twice as large as those based on F, and R- factors based on ALL data will be even larger.

### Fractional atomic coordinates and isotropic or equivalent isotropic displacement parameters (Å^2^)

**Table:**

	*x*	*y*	*z*	*U*_iso_*/*U*_eq_	
C1	0.8357 (5)	−0.0050 (5)	0.4189 (3)	0.0298 (10)	
H1	0.7730	−0.0137	0.4764	0.036*	
C2	0.8690 (5)	−0.1487 (5)	0.3753 (3)	0.0278 (10)	
C3	0.9556 (5)	−0.1377 (5)	0.2879 (3)	0.0284 (10)	
H3	0.9797	−0.2302	0.2574	0.034*	
C4	1.0077 (5)	0.0126 (5)	0.2442 (3)	0.0281 (10)	
C5	0.9753 (5)	0.1526 (5)	0.2963 (3)	0.0268 (10)	
C6	1.0337 (6)	0.3077 (6)	0.2576 (4)	0.0335 (11)	
H6	1.0133	0.3995	0.2916	0.040*	
C7	1.1193 (6)	0.3232 (6)	0.1711 (4)	0.0408 (12)	
H7	1.1604	0.4260	0.1473	0.049*	
C8	1.1461 (6)	0.1846 (6)	0.1173 (4)	0.0413 (12)	
H8	1.2010	0.1959	0.0567	0.050*	
C9	1.0927 (6)	0.0338 (6)	0.1529 (4)	0.0388 (12)	
H9	1.1125	−0.0564	0.1166	0.047*	
C10	0.8127 (5)	−0.3042 (5)	0.4268 (3)	0.0297 (10)	
C11	0.5510 (6)	0.8739 (5)	0.6646 (3)	0.0298 (10)	
H11	0.6006	0.8810	0.6021	0.036*	
C12	0.5331 (5)	1.0201 (5)	0.7144 (3)	0.0271 (10)	
C13	0.4575 (5)	1.0106 (5)	0.8051 (3)	0.0286 (10)	
H13	0.4414	1.1043	0.8391	0.034*	
C14	0.4036 (6)	0.8582 (6)	0.8472 (3)	0.0309 (10)	
C15	0.4289 (5)	0.7163 (5)	0.7920 (4)	0.0298 (10)	
C16	0.3802 (6)	0.5640 (6)	0.8339 (4)	0.0391 (12)	
H16	0.3952	0.4710	0.7984	0.047*	
C17	0.3109 (7)	0.5526 (7)	0.9267 (4)	0.0519 (14)	
H17	0.2809	0.4517	0.9544	0.062*	
C18	0.2839 (7)	0.6908 (7)	0.9811 (4)	0.0540 (15)	
H18	0.2359	0.6804	1.0440	0.065*	
C19	0.3277 (6)	0.8391 (6)	0.9421 (4)	0.0413 (12)	
H19	0.3075	0.9294	0.9781	0.050*	
C20	0.5961 (6)	1.1778 (5)	0.6657 (4)	0.0302 (10)	
N1	0.8868 (4)	0.1405 (4)	0.3842 (3)	0.0287 (8)	
N2	0.5033 (5)	0.7289 (4)	0.6994 (3)	0.0306 (9)	
O1	0.7023 (4)	−0.3102 (4)	0.4894 (3)	0.0389 (8)	
O2	0.8812 (4)	−0.4181 (4)	0.4039 (2)	0.0372 (8)	
O3	0.5866 (5)	1.3064 (4)	0.7133 (3)	0.0479 (9)	
O4	0.6565 (4)	1.1679 (4)	0.5802 (3)	0.0428 (9)	
Ag1	0.79527 (5)	0.35203 (4)	0.48083 (3)	0.03910 (14)	
Ag2	0.60900 (5)	0.52274 (4)	0.61650 (3)	0.04425 (15)	

### Atomic displacement parameters (Å^2^)

**Table:**

	*U*^11^	*U*^22^	*U*^33^	*U*^12^	*U*^13^	*U*^23^
C1	0.034 (2)	0.027 (2)	0.031 (2)	0.0094 (19)	0.0114 (19)	0.0067 (19)
C2	0.030 (2)	0.025 (2)	0.030 (2)	0.0087 (18)	0.0055 (18)	0.0059 (19)
C3	0.038 (2)	0.022 (2)	0.027 (2)	0.0122 (18)	0.0074 (18)	−0.0037 (18)
C4	0.030 (2)	0.027 (2)	0.028 (2)	0.0090 (18)	0.0047 (18)	0.0029 (19)
C5	0.028 (2)	0.027 (2)	0.028 (2)	0.0109 (18)	0.0063 (18)	0.0026 (18)
C6	0.039 (2)	0.023 (2)	0.041 (3)	0.0095 (19)	0.011 (2)	0.009 (2)
C7	0.049 (3)	0.033 (3)	0.043 (3)	0.010 (2)	0.013 (2)	0.014 (2)
C8	0.052 (3)	0.042 (3)	0.034 (2)	0.013 (2)	0.020 (2)	0.008 (2)
C9	0.049 (3)	0.033 (3)	0.039 (3)	0.015 (2)	0.015 (2)	0.004 (2)
C10	0.035 (2)	0.026 (2)	0.028 (2)	0.0075 (19)	0.0043 (19)	0.0004 (19)
C11	0.039 (2)	0.025 (2)	0.028 (2)	0.0107 (19)	0.0117 (19)	0.0057 (19)
C12	0.031 (2)	0.022 (2)	0.031 (2)	0.0085 (18)	0.0056 (18)	0.0040 (19)
C13	0.039 (2)	0.022 (2)	0.028 (2)	0.0117 (19)	0.0066 (18)	0.0001 (18)
C14	0.037 (2)	0.028 (2)	0.030 (2)	0.0104 (19)	0.0079 (19)	0.0065 (19)
C15	0.030 (2)	0.025 (2)	0.036 (2)	0.0079 (18)	0.0079 (19)	0.0067 (19)
C16	0.053 (3)	0.027 (2)	0.039 (3)	0.011 (2)	0.014 (2)	0.006 (2)
C17	0.069 (3)	0.040 (3)	0.050 (3)	0.012 (2)	0.019 (3)	0.017 (2)
C18	0.067 (3)	0.053 (3)	0.045 (3)	0.014 (3)	0.029 (3)	0.014 (3)
C19	0.053 (3)	0.037 (3)	0.036 (3)	0.013 (2)	0.013 (2)	0.004 (2)
C20	0.038 (2)	0.021 (2)	0.034 (2)	0.0117 (19)	0.008 (2)	0.0045 (19)
N1	0.0348 (19)	0.021 (2)	0.031 (2)	0.0072 (16)	0.0088 (16)	0.0006 (16)
N2	0.042 (2)	0.023 (2)	0.031 (2)	0.0113 (16)	0.0147 (17)	0.0053 (16)
O1	0.0514 (19)	0.0296 (18)	0.0437 (19)	0.0174 (15)	0.0238 (16)	0.0104 (15)
O2	0.0490 (18)	0.0205 (17)	0.0459 (19)	0.0114 (14)	0.0182 (16)	0.0036 (15)
O3	0.078 (2)	0.0253 (18)	0.046 (2)	0.0173 (17)	0.0210 (18)	0.0058 (16)
O4	0.061 (2)	0.0273 (18)	0.046 (2)	0.0123 (15)	0.0296 (17)	0.0072 (15)
Ag1	0.0547 (3)	0.0202 (2)	0.0470 (3)	0.01195 (18)	0.01993 (19)	0.01007 (18)
Ag2	0.0681 (3)	0.0223 (2)	0.0496 (3)	0.0169 (2)	0.0257 (2)	0.01112 (19)

### Geometric parameters (Å, º)

**Table:**

C1—N1	1.315 (5)	C12—C20	1.501 (6)
C1—C2	1.410 (6)	C13—C14	1.411 (6)
C1—H1	0.9300	C13—H13	0.9300
C2—C3	1.374 (6)	C14—C19	1.416 (6)
C2—C10	1.495 (6)	C14—C15	1.433 (6)
C3—C4	1.404 (6)	C15—N2	1.381 (5)
C3—H3	0.9300	C15—C16	1.406 (6)
C4—C9	1.412 (6)	C16—C17	1.363 (7)
C4—C5	1.424 (6)	C16—H16	0.9300
C5—N1	1.386 (5)	C17—C18	1.407 (8)
C5—C6	1.416 (6)	C17—H17	0.9300
C6—C7	1.359 (6)	C18—C19	1.355 (7)
C6—H6	0.9300	C18—H18	0.9300
C7—C8	1.405 (7)	C19—H19	0.9300
C7—H7	0.9300	C20—O3	1.245 (5)
C8—C9	1.361 (7)	C20—O4	1.252 (5)
C8—H8	0.9300	Ag1—N1	2.429 (3)
C9—H9	0.9300	Ag1—O2^i^	2.219 (3)
C10—O1	1.246 (5)	Ag1—O4^ii^	2.220 (3)
C10—O2	1.261 (5)	Ag1—Ag2	2.9468 (6)
C11—N2	1.310 (5)	Ag2—N2	2.373 (3)
C11—C12	1.410 (6)	Ag2—O1^i^	2.282 (3)
C11—H11	0.9300	Ag2—O3^ii^	2.258 (3)
C12—C13	1.364 (6)	Ag2—Ag2^iii^	3.3099 (10)
			
N1—C1—C2	124.9 (4)	N2—C15—C14	120.5 (4)
N1—C1—H1	117.6	C16—C15—C14	119.4 (4)
C2—C1—H1	117.6	C17—C16—C15	120.0 (5)
C3—C2—C1	117.8 (4)	C17—C16—H16	120.0
C3—C2—C10	123.0 (4)	C15—C16—H16	120.0
C1—C2—C10	119.2 (4)	C16—C17—C18	121.1 (5)
C2—C3—C4	120.3 (4)	C16—C17—H17	119.4
C2—C3—H3	119.9	C18—C17—H17	119.4
C4—C3—H3	119.9	C19—C18—C17	120.3 (5)
C3—C4—C9	124.0 (4)	C19—C18—H18	119.9
C3—C4—C5	117.9 (4)	C17—C18—H18	119.9
C9—C4—C5	118.1 (4)	C18—C19—C14	120.8 (5)
N1—C5—C6	119.0 (4)	C18—C19—H19	119.6
N1—C5—C4	121.5 (4)	C14—C19—H19	119.6
C6—C5—C4	119.5 (4)	O3—C20—O4	125.6 (4)
C7—C6—C5	120.4 (4)	O3—C20—C12	118.1 (4)
C7—C6—H6	119.8	O4—C20—C12	116.2 (4)
C5—C6—H6	119.8	C1—N1—C5	117.6 (4)
C6—C7—C8	120.2 (5)	C1—N1—Ag1	113.6 (3)
C6—C7—H7	119.9	C5—N1—Ag1	128.7 (3)
C8—C7—H7	119.9	C11—N2—C15	118.1 (4)
C9—C8—C7	120.8 (5)	C11—N2—Ag2	115.5 (3)
C9—C8—H8	119.6	C15—N2—Ag2	125.0 (3)
C7—C8—H8	119.6	C10—O1—Ag2^ii^	134.4 (3)
C8—C9—C4	120.9 (4)	C10—O2—Ag1^ii^	117.0 (3)
C8—C9—H9	119.6	C20—O3—Ag2^i^	115.2 (3)
C4—C9—H9	119.6	C20—O4—Ag1^i^	133.5 (3)
O1—C10—O2	125.3 (4)	O2^i^—Ag1—O4^ii^	161.54 (11)
O1—C10—C2	117.1 (4)	O2^i^—Ag1—N1	107.52 (12)
O2—C10—C2	117.6 (4)	O4^ii^—Ag1—N1	90.23 (12)
N2—C11—C12	125.2 (4)	O2^i^—Ag1—Ag2	88.38 (8)
N2—C11—H11	117.4	O4^ii^—Ag1—Ag2	73.44 (8)
C12—C11—H11	117.4	N1—Ag1—Ag2	162.81 (9)
C13—C12—C11	117.9 (4)	O3^ii^—Ag2—O1^i^	157.36 (12)
C13—C12—C20	123.0 (4)	O3^ii^—Ag2—N2	111.17 (12)
C11—C12—C20	119.1 (4)	O1^i^—Ag2—N2	90.66 (11)
C12—C13—C14	119.9 (4)	O3^ii^—Ag2—Ag1	85.21 (8)
C12—C13—H13	120.1	O1^i^—Ag2—Ag1	72.49 (8)
C14—C13—H13	120.1	N2—Ag2—Ag1	162.51 (9)
C13—C14—C19	123.2 (4)	O3^ii^—Ag2—Ag2^iii^	119.91 (9)
C13—C14—C15	118.4 (4)	O1^i^—Ag2—Ag2^iii^	58.54 (9)
C19—C14—C15	118.4 (4)	N2—Ag2—Ag2^iii^	100.68 (9)
N2—C15—C16	120.1 (4)	Ag1—Ag2—Ag2^iii^	74.839 (19)

Symmetry codes: (i) *x*, *y*+1, *z*; (ii) *x*, *y*−1, *z*; (iii) −*x*+1, −*y*+1, −*z*+1.
